# Heritability of Oral Microbiota and Immune Responses to Oral Bacteria

**DOI:** 10.3390/microorganisms8081126

**Published:** 2020-07-27

**Authors:** Anders Esberg, Simon Haworth, Ralf Kuja-Halkola, Patrik K.E. Magnusson, Ingegerd Johansson

**Affiliations:** 1 Department of Odontology, Umeå University, 901 87 Umeå, Sweden; ingegerd.johansson@umu.se; 2 Medical Research Council Integrative Epidemiology Unit, Department of Population Health Sciences, Bristol Medical School, University of Bristol, Bristol BS8 2BN, UK; simon.haworth@bristol.ac.uk; 3 Bristol Dental School, University of Bristol, Bristol BS1 2LY, UK; 4 Department of Medical Epidemiology and Biostatistics, Karolinska Institutet, 171 77 Stockholm, Sweden; ralf.kuja-halkola@ki.se (R.K.-H.); patrik.magnusson@ki.se (P.K.M.)

**Keywords:** heritability, saliva, microbiota, 16S rDNA, antibody, immunoblotting

## Abstract

Maintaining a symbiotic oral microbiota is essential for oral and dental health, and host genetic factors may affect the composition or function of the oral microbiota through a range of possible mechanisms, including immune pathways. The study included 836 Swedish twins divided into separate groups of adolescents (*n* = 418) and unrelated adults (*n* = 418). Oral microbiota composition and functions of non-enzymatically lysed oral bacteria samples were evaluated using 16S rRNA gene sequencing and functional bioinformatics tools in the adolescents. Adaptive immune responses were assessed by testing for serum IgG antibodies against a panel of common oral bacteria in adults. In the adolescents, host genetic factors were associated with both the detection and abundance of microbial species, but with considerable variation between species. Host genetic factors were associated with predicted microbiota functions, including several functions related to bacterial sucrose, fructose, and carbohydrate metabolism. In adults, genetic factors were associated with serum antibodies against oral bacteria. In conclusion, host genetic factors affect the composition of the oral microbiota at a species level, and host-governed adaptive immune responses, and also affect the concerted functions of the oral microbiota as a whole. This may help explain why some people are genetically predisposed to the major dental diseases of caries and periodontitis.

## 1. Introduction

Bacterial communities in the oral cavity are among the most dense and diverse in the human body [1,2]. Studies investigating the role of oral bacteria in maintaining oral health or the development of dental diseases initially focused on single species, but now increasingly examine the concerted microbiome using DNA-based methods. Although the general understanding from the latter studies is that resilient and diverse profiles of the tooth or saliva bacterial communities are beneficial for health [3,4], there is also agreement that the variation in host genetics and environmental exposures affect disease variation [5,6,7].

The main colonization of the gastro-intestinal canal takes place after birth and stabilizes into niche-specific polymicrobial biofilms during childhood [8]. Several studies demonstrate the importance of parental influence as one important determinant in forming the bacterial communities in the mouth and the gut at early age [9,10]. This might be due to both within-family transmission and genetic predisposition to bacteria acquisition. Sugar intake is known to affect the oral microbiome composition, whereas the relative importance of other environmental and host gene variations on variation and architecture of the oral microbiota are not clearly identified [11].

The role of host genetics in oral microbiota composition is supported by early studies where monozygotic twin pairs had greater concordance for targeted species or microbiota related traits than dizygotic twins [12,13,14,15]. Later, DNA-based studies of saliva and tooth biofilm microbiota in twins supported both genetic and environmental effects, but with inconclusive results. Some authors suggest that environmental factors have a stronger effect on the composition of the supragingival microbiota than genetic factors [16,17,18]. Others argue that the genetic factors are important [19,20,21] and report significant heritable additive genetic variance for *Prevotella pallens*, *Corynebacterium durum,* and species in genera, including the *Veillonella*, *Pasteurellaceae*, and *Abiotrophia* genera [21].

Understanding the forces which shape and maintain the oral microbial communities is key to understand their role in oral, and possibly general health. The architecture of the bacterial communities is usually characterized by describing the abundance and types of bacteria in the habitat. A different approach is to assess microbial community functions, i.e., metabolic and other activities. Recent findings indicate that oral microbiotas with different compositions may still have similar functions and represent similar bacterial ecosystems [22]. Hence, estimating bacteria community functions may provide an alternative way to characterize the oral microbiota, which is more biologically and functionally relevant for oral diseases or host interactions such as maturation of immunity. At this point, it may be concluded that there is support for both genetic and environmental influences on taxonomic aspects of the oral microbiota but the relative importance is not unanimously defined, especially not at the species level, and there is little data on the potential heritability of functional aspects and associations with immune responses to common bacteria in the mouth.

The aims of the present study were to estimate the heritability of (i) measures of oral microbiota taxonomic composition and function derived from 16S rDNA amplicon sequencing of saliva extracted DNA and (ii) antibody profiles to a panel of 19 oral bacterial species. This was done in (i) a cohort of 418 young twins, and (ii) a cohort of 418 adult twins from the Swedish Twin Registry [23]. The main conclusions from the study were that both additive genetic and unshared environmental factors shape the taxonomic profile and functional aspects of the saliva microbiota, as well as antibody responses to oral bacteria.

## 2. Materials and Methods

### 2.1. Study Participants

Two independent participant groups were recruited from existing sub-studies of the Swedish Twin Registry (STR, https://ki.se/en/research/swedish-twin-registry-for-researchers). The first group included 418 adolescents who had participated in the Child and Adolescent Twin Study in Sweden (CATSS [24]), and for whom saliva had been collected and DNA extracted [23]. For the present study, the twins were born between 1993 and 2001, and the saliva samples were collected between 2006 and 2017. The selected twins represented monozygotic twins (MZ) (*n* = 71 pairs) and dizygotic twins of the same sex (DZ-SS) (*n* = 68 pairs) and of the opposite sex (DZ-OS) (*n* = 70 pairs).

The second group was comprised of 418 adults who had participated in the Screening Across the Lifespan Twin Study (SALT [25]), which is also part of STR. In SALT, all Swedish twins born between 1896 and 1958 were invited. For the present study, the twins were born between 1917 and 1958, and the blood was collected between 2004 and 2008.

Information on dental status was collected from the Swedish quality register on caries and periodontitis, SKaPa, (www.skapareg.se/). For people with records from more than one visit, information was taken from the dental visit, which was most contemporaneous with participation in CATSS or SALT.

### 2.2. Ethics Statement

The CATSS study received ethical approval from the Karolinska Institutet Ethical Review Board (CATSS-15 Dnr: 2009/739-31/5) and saliva collection and DNA extraction in Dnr: 03-672, 2010/597-31/1, 2016/2135-31, and 2018/960-31/2). Data collection in SALT was approved by the Research Ethics Committee at Karolinska Institutet (Dnr: 2007/644-31), and informed consent was obtained from all participants. The present study was approved by the Regional Ethical Committee at Umeå University (Dnr: 2018/232-31 and 2018/311-32M).

### 2.3. Zygosity Determination

Zygosity was determined by genotyping of 46 single nucleotide polymorphism (SNPs), and the results were evaluated using an algorithm allowing for potential laboratory errors. The full protocol has previously been published [26].

### 2.4. Saliva Collection and DNA Extraction for Microbiota Determination

The parents and participating adolescents were contacted by telephone with information about saliva collection. Collection kits (Oragene OG-500, DNA Genotek Inc., Ottawa, ON, Canada) with a unique barcode were sent to the participants’ homes where the twins collected saliva according to the instructions provided by the company. Briefly, the participants were instructed not to eat, drink, or chew gum for 30 min before sampling, and to spit saliva into the funnel on top of a test tube until approximately 2 mL were collected. The funnel was removed, the tube capped and shaken for 5 sec. The samples were mailed back to the project using the standard postal system. The Oragene tube contains a pre-extraction preservation buffer that stabilizes the sample at room temperature and which is compatible with DNA high-throughput sequencing [23].

DNA was extracted from saliva at the Karolinska Institutet (KI) Biobank (Stockholm, Sweden) using the automatic systems of Puragene (Qiagen AB, Sollentuna, Sweden) or Chemagen (PerkingElmer, Hägersten, Sweden). The extracted DNA was stored at −80 °C.

### 2.5. Validation of DNA Extraction

The kit used for DNA extraction for the Swedish Twin Registry samples lacked the additional enzymes commonly used to facilitate breakage of the cell walls of more hard-to-break bacteria. To estimate how this would affect the yield, a validation analysis was performed where seven saliva samples and three mock samples of known bacterial species were extracted at the KI Biobank and at our laboratory in Umeå using our routine method, i.e., the GenElute Bacterial Genomic DNA (Sigma-Aldrich, St. Louis, MO, USA) with Proteinase K and RNase and additional lysozyme and mutanolysin [22]. Sequencing was done in two independent runs at the Illumina MiSeq platform as described previously [22] and followed the same bioinformatic process as used for the twin samples. The known bacterial mixtures were created from combinations of 17 species (Table S1).

### 2.6. Illumina Sequencing

Bacterial 16S rDNA amplicons were generated from the v3–v4 regions by PCR using fusion primers with 341F (ACGGGAGGCAGCAG) forward and 806R (GGACTACHVGGGTWTCTAAT) reverse primers from saliva and a mock community extracted DNA, as described by Caporaso [27]. The 17 mock community members are shown in Table S1. Equimolar libraries were pooled and purified using AMPure XP beads (Beckman Coulter, Stockholm, Sweden) and sequenced using the Illumina Miseq platform. The samples were analyzed in five runs, and each run contained an addition of 5% PhiX (Illumina, Stockholm, Sweden), two mock samples (Table S1), and two negative controls (ultra-pure water).

Obtained sequences were de-multiplexed, pair-end reads fused, primers, ambiguous, and chimeric sequences and PhiX were removed, and amplicon sequence variants (ASVs) were obtained using the open-source software package DADA2 in the QIIME2 next-generation microbiome bioinformatics platform (https://qiime2.org) [28,29]. ASVs were taxonomically classified against the expanded Human Oral Microbiome Database (eHOMD) (http://www.homd.org) [29,30]. Only ASVs with at least two reads and 98.5% identity with a named species or unnamed phylotype in eHOMD were retained, and those with the same Human Microbial Taxon (HMT) ID number were aggregated. The negative control yielded <25 sequences, and the positive control (mock communities) was correct for representative sequences with ≥20. Accordingly, the comparisons were based upon taxa with >25 reads. For simplicity, all named species or unnamed phylotypes are referred to as species.

### 2.7. Blood Collection and Serum Antibody Profile Determination

Twins in the SALT study had been asked to visit their local health care center and provide blood for serum preparation and DNA extraction for zygosity determination in a follow-up study called TwinGene [23]. Serum was prepared according to clinical routine and stored in aliquots. For the present study, blood samples were collected between 2004 and 2008, and serum was stored at −80 °C for 12–16 years before analysis.

For the antibody detection, bacterial strains were grown for 48–72 h on blood, Rogosa, or chocolate blood agar plates in aerobic (with 5% CO_2_) or anaerobic conditions at 37 °C, as indicated in Table S1. Bacteria were harvested using sterilized cotton swabs, resuspended and washed twice in 50 mM Tris-HCl, pH 7.5, 150 mM NaCl (TBS), and adjusted to an optical density of 1.0 at 600 nm before being stored at −80 °C in 500 µL aliquots until used.

Antibody detection was done using a multiplex immunoblotting assay in a checkerboard device, as previously described [31,32]. Briefly, a *nitrocellulose membrane (*Amersham™ Protran^®^ GE10600003, Merck, Solna, Sweden), which had been pre-wet (18 Ω Milli-Q Biocel, Merck Solna, Sweden) and equilibrated in TBS for 10 min, was loaded in a Miniblotter device (Miniblotter 45MN, Interchime, Montlucon Cedex, France) according to the manufacturer’s instructions. Bacterial suspensions (150 µL), positive controls (Protein A, P6031, Merck, Solna, Sweden) at 0.0051–1 µg/mL concentration and negative controls (TBS) were loaded into the lanes of the Miniblotter, and the gasket was sealed with adhesive film to avoid evaporation. The device was rotated slowly for 1 h at room temperature followed by overnight incubation at 4 °C on a balanced table. The liquids were removed from the lanes by vacuum suction and washed with 500 µL TBS with 0.1% Tween 20 (TBS-T) with the membrane still in the Miniblotter. Then the membrane was removed and rinsed 3 × 1 min in TBS-T prior to blocking in TBS-T containing 5% blocking reagent (ECL™ Advance Blocking Reagent, GERPN418, Merck) for 1 h under rotation at room temperature. The Miniblotter was re-assembled, and serum diluted 1:500 in TBS was applied perpendicular to the bacteria, sealed with adhesive film, and slowly rotated for 1 h at room temperature. The liquids were removed by vacuum suction and washed once with 500 µL TBS-T. The membrane was removed and rinsed 3 × 1 min in TBS-T before treatment with 0.3% H_2_O_2_ for 10 min (H_2_O_2_, H1009, Merck, Solna, Sweden) to reduce endogenous bacterial peroxidase activity. This was followed by rinsing and washing of the membrane 1 × 15 min, 3 × 5 min using TBS-T before incubation with an Anti-Human-IgG-Fab peroxidase labeled secondary antibody (Anti-Human-IgG-Fab, A0293, Merck, Solna, Sweden) diluted in TBS-T with 5% blocking powder for 1 h at room temperature under slow rotation. Finally, the membrane was rinsed twice, washed 1 × 15 min, 3 × 5 min using TBS-T before signal development (ECL™ Prime Western Blotting Detection Reagent, GERPN2236, Merck). Signals were detected using the ChemiDoc^TM^ XRS + System (BioRad, Solna, Sweden).

### 2.8. Statistics

#### 2.8.1. Basic Descriptive Analysis

Descriptive statistics are presented as means with 95% confidence intervals (CI) or percentages depending on the variable characteristics. Caries status was retrieved from electronic records at the participants’ dental clinics and was summarized using the decayed, missing, and filled tooth surfaces index for permanent teeth (DMFS), which is a measure of the cumulative caries experience. Group differences were tested with non-parametric tests unless otherwise stated.

#### 2.8.2. Generation of Standardized Abundance Variables and Definition of Detection

The reads for quality filtered and HMT aggregated taxa were standardized to the level of the sample with fewest reads (20,016 reads), and then transformed by inverse hyperbolic sine transformation. Inverse hyperbolic sine transformation defines log values, including for zero-values, which are prevalent for many species. Detection (carriage) of a species was set to having ≥1 read for the species.

#### 2.8.3. Intraclass Correlation Comparisons

Intraclass Correlation Coefficients (ICCs) were used to estimate the variance shared between the twins compared to total variance, applying the, alpha model and one-way random effects. Fleiss’ kappa was used to estimate twin pair species concurrence compared to that expected by chance. Kernel density was used to estimate the probability density of ICC estimates.

#### 2.8.4. Predicted Functions

Potential molecular functions of the saliva microbiota were predicted using the Phylogenetic Investigation of Communities by Reconstruction of Unobserved States (PICRUSt2 [33] plugin for QIIME2 and converted to functions via the Kyoto Encyclopedia of Genes and Genomes (KEGG) Orthology database (KO) (https://www.genome.jp/kegg/ko) [33,34]. Falconer’s formula was used to estimate the relative contribution of genetics vs. environment for a variation in predicted functions based on the ICC difference between the monozygotic (ICCmz) and dizygotic (ICCdz) twins (2 × (ICCmz-ICCdz)) [35].

#### 2.8.5. Estimating Heritability

Quantitative twin models [36] were fitted to estimate the heritability of (a) each species-level detection trait (for species detected in 5–95% of samples), (b) each standardized abundance count (for species detected in 5–100% of samples), (c) a subset of KO predictions, where Falconer’s formula indicated a heritable component, and d) all quantitative measures of serum antibody response. These models (referred to as the ACE models) decompose the total variation in a trait into variation due to three different components. Variation due to additive genetic effects is termed variance component A. Variation due to shared environmental factors that affect both twins in a pair equally is termed variance component C. Hence, both A and C may contribute to the correlation in a trait within twin pairs. Monozygotic twins share ~100%, but in dizygotic twin pairs on average only 50% of the segregating DNA alleles, which allows the models to distinguish between components A and C. Finally, the variance in a trait, which is not explained by either of these components can be thought to arise from a mixture of non-shared environmental factors which only affect one twin in a pair and measurement error. This is termed variance component E. Models included adjustment for the birth year (linear), sex and sequencing batch (for adolescents), or antibody batch (for adults), and were fitted in the OpenMx version 2.17 [37] implemented in R (version 3.6.3). The results are only reported for models which converged successfully, and *p*-values (based on Z test, where Z was defined as the squared variance component divided by SE for the squared variance component) for variance components were adjusted using a Bonferroni correction on the basis of 181 approximately independent tests for species detection and abundance analyses, nine approximately independent tests for estimated functions, and three approximately independent tests for antibody profile (see Appendix A for estimation of multiple testing burden). Since the detection traits were coded as a binary outcome (detection or no detection), the analysis of detection traits used a liability-threshold model which assumes a non-observed underlying continuous normally distributed liability, and that detection is observed if the liability is above an estimated threshold. In this model, the association between twins is the correlation between the underlying liabilities, equivalent to a so-called tetrachoric correlation, inferred by concordance and discordance of detection across twins [38]. Analysis of all other traits was carried out on the observed scale. All estimates of A, C, and E, are presented as standardized variance components, i.e., the proportion of total variance in the trait attributed to that component.

## 3. Results

### 3.1. Study Participants

We analyzed the saliva microbiota from 418 twins (71 monozygotic pairs (MZ), 68 same-sex, and 70 opposite sex dizygotic pairs (DZ-SS and DZ-OS, respectively) born between 1993 and 2001. Mean DNA storage time was 10.5 years in all three groups (Table 1). The proportion of female and male participants was similar in the groups, as was the caries status (DMFS scores) (Table 1).

We also analyzed serum antibody profiles to 19 oral bacterial species from blood collected between 2004 and 2008. The samples were from 418 adult twins (70 MZ pairs, 69 DZ-SS, and 70 DZ-OS pairs) born between 1917 and 1958. The serum storage time (mean 14 years), participant age (mean 64 years), and cumulative caries status were largely similar in the three adult zygosity groups (Table 1).

### 3.2. Overall Sequencing Results

Amplicon sequencing of the v3–v4 variable regions of the 16S rRNA gene from saliva DNA from the 418 adolescent twins yielded 73,665,719 reads, which after merging, trimming, denoising, and removal of potential chimeric sequences, retained an average of 64,273 (min, max, 20,144, 113,144) paired-end reads per sample. The filtered reads corresponded to 8037 ASVs of which 5% did not match at all, 28% did not meet the criteria of 98.5% identity, and 63% matched to a species or unnamed phylotype at 98.5% identity in the eHOMD 16S rRNA gene database, and were represented by ≥25 reads. These ASVs represented 406 named or unnamed species in 11 phyla and 109 genera.

**Figure 1:**
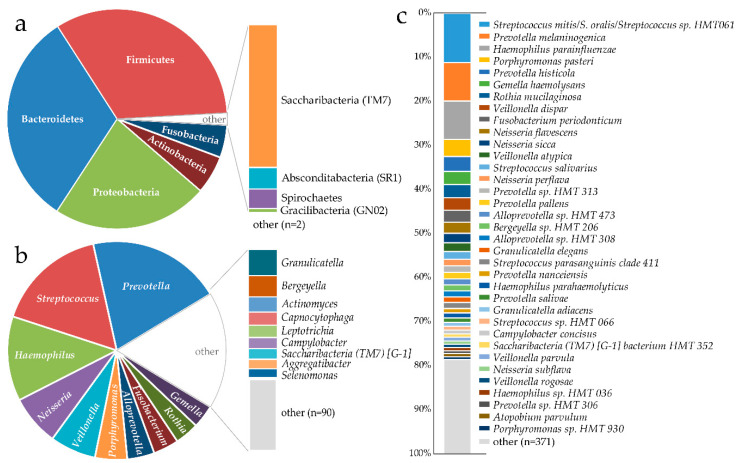
Pie and bar charts illustrating proportions of (**a**) identified phyla, (**b**) top identified genera, and (**c**) top eHOMD matched species.

Detected phyla were in descending order Firmicutes (33.2%), Bacteroidetes (31.7%), Proteobacteria (22.9%), Actinobacteria (5.7%), Fusobacteria (4.8%), Saccharibacteria (TM7) (1.3%), Absconditabacteria (SR1) (0.20%), Spirochaetes (0.18%), Gracilibacteria (GN02) (<0.01%), Cyanobacteria (<0.01%), and Synergistetes (<0.01%) (Figure 1a). The top 10 genera were in descending order *Prevotella* (19.8%), *Streptococcus* (16.5%), *Haemophilus* (12.6%), *Neisseria* (7.5%), *Veillonella* (6.8%), *Porphyromonas* (4.8%), *Alloprevotella* (4.0%), *Fusobacterium* (3.8%), *Rothia* (3.6%), and *Gemella* (3.5%) (Figure 1b). The top ten species or species aggregates were *Streptococcus mitis/Streptococcus oralis/Streptococcus* HMT061 (11.2%), *Prevotella melaninogenica* (8.8%), *Haemophilus parainfluenzae* (8.7%), *Porphyromonas pasteri* (3.8%), *Prevotella histicola* (3.4%), *Gemella haemolysans* (3.1%), and *Rothia mucilaginosa* (3.0%), *Veillonella dispar* (2.8%), *Fusobacterium periodonticum* (2.7%), and *Neisseria flavescens* (2.5%) (Figure 1c).

### 3.3. Species Retrieval by DNA Extraction Method

DNA from seven saliva samples and one mock bacteria sample was extracted by the method used for the twin samples, and by an oral bacteria DNA optimized method (see methods section) and was run in two independent runs. The mean Spearman correlation coefficient between yields expressed as standardized bacterial counts for the seven saliva samples was 0.66 with a range from 0.51 to 0.86. The corresponding correlation for the mock species was 0.91, with a range from 0.82 to 1.0. All bacterial species in the mock mixtures were recognized by both DNA extraction methods, but the standardized counts were markedly lower for the method used for the twin samples for some Gram-positive species, including *S. mutans, S. sanguinis, A. odontolyticus*, *C. matruchotii*, *L. fermentum*, *S. parasanguinis*, but not for Gram-positive species in *Bifidobacteria, Gemella, Rothia, Veillonella*, or tested Gram-negative species. The results from the independent duplicate runs were virtually identical.

### 3.4. Overall Saliva Microbiota Diversity by Twin Zygosity

**Figure 2:**
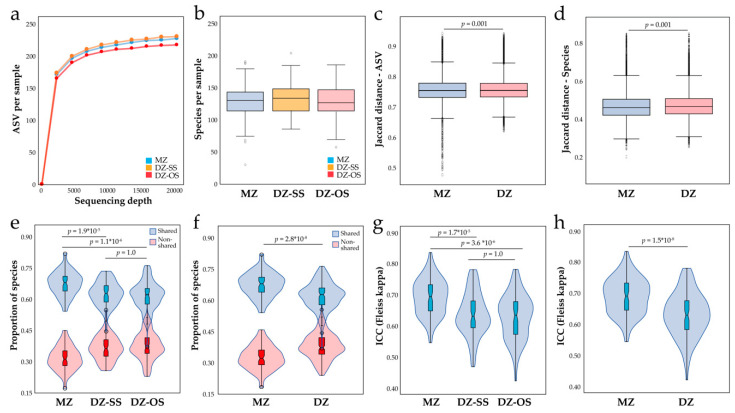
Overall microbiota comparisons between MZ and DZ groups. (**a**) alpha diversity of MZ, DZ-SS, and DZ-OS twin groups based on rarefaction curves of identified ASVs per sample, and (**b**) number of eHOMD-identified species in the same groups. (**c**) Box plot of Jaccard diversity index based on (**c**) ASV profiles and (**d**) eHOMD species detection. Violin plots showing (**e**) pair-wise proportions of shared and non-shared species in the three zygosity groups and (**f**) MZ and merged DZ twin groups. Intraclass qualitative estimation agreement based on Fleiss’ kappa values for (**g**) MZ, DZ-SS, and DZ-OS twin groups and (**h**) MZ and merged DZ groups. The Mann–Whitney U tests were used for group comparisons.

The number of ASVs per twin (overall mean 64,273 (95% CI 62,620, 65,926)) did not differ between the three zygosity groups (*p*_ANOVA_ = 0.27), nor did the alpha diversity scores ASVs per sample (*p* = 0.77) and Shannon index (*p* = 0.68) (Figure 2a). Similarly, no difference was seen between the three zygosity groups for the total number of eHOMD identified species (overall median 133, (95% CI 130, 134), *p*_ANOVA_ = 0.37) (Figure 2b).

The ASV-based Jaccard distances (beta-diversity from the presence or not of an ASV) was significantly lower among MZ compared to DZ zygosity twins (*p* = 0.001, multivariate PERMANOVA analysis, Bonferroni-corrected *p*-values, 9999 permutations) (Figure 2c). This was also seen for MZ versus each of the DZ groups (both *p* = 0.001) but not between DZ-SS and DZ-OS. Similarly, the Jaccard distance of eHOMD detected species was lower among the MZ than DZ twins (*p* = 0.001, Figure 2d), and DZ-SS and DZ-OS twins separately (*p* = 0.031 and *p* = 0.021, respectively), but not between DZ-SS and DZ-OS twins (*p* = 0.513). The microbiota profile did not differ between the three zygosity groups at the phylum, class, family, order, or genus levels.

Overall, the twins shared 62.4% (95% CI 61.4, 63.4) of the 406 species, with a significantly higher proportion of shared species among the MZ compared with DZ-SS or DZ-OS twins (*p* = 1.9 × 10^−5^ and *p* = 1.1 × 10^−5^, respectively, Figure 2e) or DZ together (*p* = 2.8 × 10^−8^, Figure 2f). No difference was seen between the two DZ groups (*p* = 1.0). Fleiss’ kappa confirmed a higher ICC degree of agreement in the MZ group compared to each of the two DZ groups separately (Figure 2g), or a merged group of all the DZ twins (Figure 2h). In concert, these results indicate a heritable influence on the oral microbiota. None of these measures differed between the sexes.

### 3.5. Intraclass Correlations of Saliva Microbiota Species in MZ versus DZ Twins

**Figure 3:**
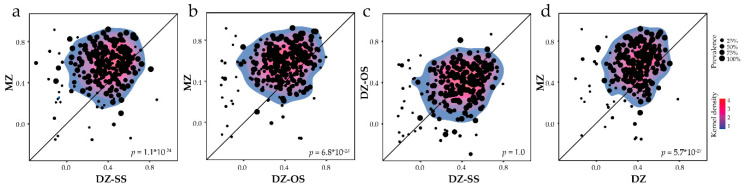
Scatter plot of intraclass correlation coefficient in MZ versus DZ twins. (**a**) bacterial species intraclass correlation coefficient (ICC) plotted for MZ versus DZ-SS twins, (**b**) MZ versus DZ-OS twins, (**c**) DZ-SS versus DZ-OS twins, and (**d**) MZ versus all DZ twins. ICC values are presented for species with a prevalence ≥5%. Each dot represents a single species with the dot-size indicating the species prevalence. The line represents the diagonal where X = Y. The Mann–Whitney U tests were used for group comparisons. Density was predicted using the Kernel estimation.

As a next step, ICCs of relative abundance for the detected species in MZ versus DZ-SS and DZ-OS and merged DZ twin groups were compared (Figure 3a–d). MZ twins had generally higher ICC than DZ-SS twins (*p* = 1.1 × 10^−24^, Figure 3a) and DZ-OS twins (*p* = 6.8 × 10^−23^, Figure 3b), whereas no difference was found between the two DZ groups (*p* = 1.0, Figure 3c). Comparison with merged DZ twins revealed a strong difference at the species level ICC (*p* = 5.7 × 10^−27^, Figure 3d) and higher taxonomic levels, i.e., genus (*p* = 3.2 × 10^−13^), family (*p* = 5.2 × 10^−6^) and order (*p* = 5.2 × 10^−6^), but not at the class (*p* = 0.11) and phylum levels (*p* = 0.21).

Based on the findings that the DZ-SS and DZ-OS groups were constantly similar in comparison with the MZ group, further comparisons were restricted to comparisons between MZ versus the merged DZ groups.

### 3.6. Heritability of Species Relative Abundance and Detection

Heritability was estimated by quantitative genetic twin modeling of A, C, and E of the relative abundance of bacterial species and of carrying a species, i.e., if the species was detected or not. For the former evaluation, species that were detected in less than 5% were excluded, and for the latter, those present in more than 95%. This left 267 species for heritability estimation of relative abundance (Table S2) and 239 species for the estimation of carrying a species (Table S3).

**Figure 4:**
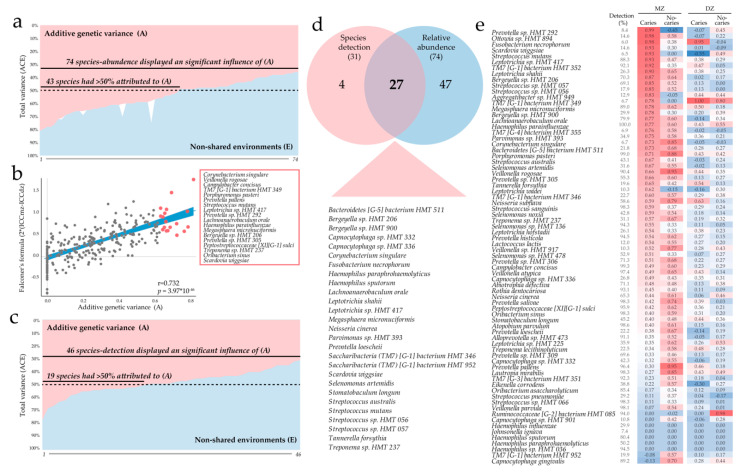
Oral microbiota heritability. (**a**) additive genetic (A) versus unique environment (E) scores for bacterial species with sex, birth-year, and Illumina run adjusted statistically significant A score for species abundance; (**b**) comparison of A scores for abundance and scores by the Falconer’s formula (2 × (ICCmz-ICCdz); (**c**) A and E score for species detection of species. Variance explained by component C in (**a**) and (**c**) is shown in white. (**d**) Venn diagram showing bacterial species with a statistically significant A score for both detection and abundance (*n* = 27); (**e**) heatmap showing ICC scores for bacterial species with a statistically significant A score by MZ and DZ and caries status (caries-free versus caries-affected)**.**

The abundance variance that could be attributed to A was statistically significant (after adjustment for sex, birth year, and sequencing run, and correction for multiple testing) for 28% (74 of 267 species) of the identified species, and of this 58%, (43 of 74 species) more than half of the variance could be attributed to A (Figure 4a and Table S2). For 77% (205 of 267 species) of the species, more than half of the variance could be attributed to E, which also includes possible error components (Figure 4a and Table S2). The results from the ACE modeling were largely consistent with estimates using Falconer’s formula on ICC from species abundances (Figure 4b). The pattern was less pronounced when detection was used, i.e., variance attributable to additive genetic variance (A) was statistically significant for 13% (31 of 239 species) and for all of these, more than half of the variance could be attributed to A (Table S3). Non-shared environmental factors (E) accounted for more than half of the variance in 58 species (Table S3). Twenty-seven species had statistically significant additive genetic variance (A) for both species relative abundance and detection (Figure 4c). Component C was generally estimated at, or close to, 0 and was statistically non-significant for >90% of the species (Table S2 and Table S3).

Taken together, the results confirm that non-shared environmental factors explain variation in bacterial detection and abundance for most species, but additive genetic host factors are important in a species-specific manner. The strongest additive genetic effects (abundance variance explained by A >0.75) were found for *Corynebacterium singular, Campylobacter concisus, Veillonella rogosae, Saccharibacteria* (TM7) [G-1] bacterium HMT 349. Among species with a statistically significant additive genetic contribution for both abundance and detection were *Streptococcus mutans* (A = 0.69 and 0.80 for abundance and detection, respectively) and *Scardovia wiggsiae* (A = 0.63 and 0.79, respectively), which are found to associate with caries in various populations, including Sweden [39,40], and *Stomatobaculum longum* (A = 0.56 and 0.76 for abundance and detection, respectively), which is found to be associated with caries development in *S. mutans* carrying-adolescent in Sweden [39] (Tables S1 and S2). Additional species with significant additive genetic effects (A) for both abundance and detection are listed in Figure 4d. To test whether caries interacts with the relationship between host genetic factors and microbiota composition, we undertook a sensitivity analysis in subsets of twin pairs with concordant caries status (caries-free or caries-affected). In possible support of interaction for *S. mutans*, strong ICC scores (0.99) were seen in MZ caries-affected young twins but not in MZ caries-free or DZ twins (Figure 4e).

### 3.7. Heritability of Predicted Functions

**Figure 5:**
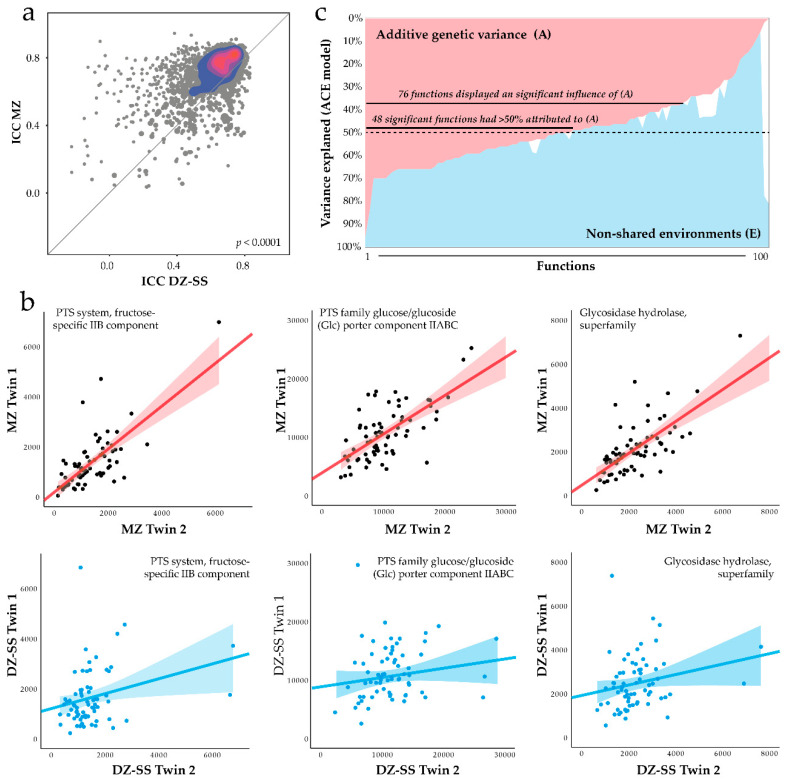
Effect of the host genotype on the microbiota predicted functions. (**a**) ICC between MZ and DZ-SS twins of the predicted functions (*n* = 3252, 100% prevalence). Density was predicted using the Kernel estimation. (**b**) 2D-scattered plots of three selected functions suggested to be prominently influenced by host additive genetic effects (A). Trend line with 95% CI is shown. (**c**) Area plot of ACE model estimation for the top 100 selected functions. Variance explained by component C is shown in white. Models were adjusted for birth year, sex, and sequencing batch, and *p*-values were adjusted using a Bonferroni correction.

We used PICRUSt [33] to generate predicted functions from the 16S rRNA gene sequences and applied ICC to visualize the overall differences and to select a panel of functions for ACE modeling. Taking all the predicted functions, which were present in all participants (*n* = 3258 KO functions), the ICC values were higher in MZ twins than DZ-SS twins, indicating that genetic factors were associated with predicted function (Figure 5a, *p <* 0.0001). The pattern was similar for the MZ twins compared to DZ-OS (*p <* 0.0001). The top 100 functions based on ICC-values (Falconer’s formula >0.76 between MZ and DZ-SS twins) were evaluated for protein-protein interaction networks (PPI) and functional enrichment using the String database. PPI analysis indicated an overall functional enrichment (*p* = 2.4 × 10^−12^) with enhancement in the KEGG pathways linked to carbohydrate metabolism, e.g., fructose and mannose metabolism (*p* = 7.4 × 10^−7^), glucose transport system (PTS) (*p* = 1.2 × 10^−5^), and starch and sucrose metabolism (*p* = 0.0077) (Figure 5b).

Next, heritability was estimated by ACE modeling of the top 100 predicted functions (Figure 5c). Additive genetic variance (A) was statistically significant for 76% of the functions, and for 63% of these (48 of 76 functions), more than half of the variance was attributed to A (Table S4). A sensitivity enrichment analysis, which selected the PPI and functional enrichment analysis of the 48 functions with over 50% attributed to A, displayed similar enrichment as the analysis which selected functions based on Falconer’s formula. Specifically, there was enrichment (overall PPI *p* = 5.4 × 10^−10^) for carbohydrate metabolism and functions associated with fructose and mannose metabolism (*p* = 1.2 × 10^−7^), the glucose transport system (PTS) (*p* = 2.8 × 10^−5^), and starch and sucrose metabolism (*p* = 0.033). A list of ICC and ACE model estimates for the 100 evaluated functions is found in Table S4.

### 3.8. Heritability of Serum Antibody Levels against a Panel of Oral Bacteria

**Figure 6:**
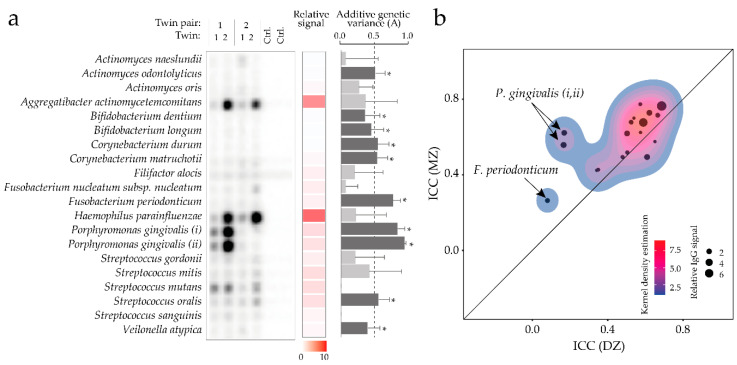
Serum IgG antibodies to oral bacteria. (**a**) show a representative checker-board analysis of two twin pairs and the negative controls (TBS) (left), heatmap for detection (middle), and bars representing additive genetic variance (A) estimates (right). Dark grey bars indicated significant attribution to additive genetic variance with the dotted line representing 50%; (**b**) scatter plot of intraclass correlation coefficient (ICC) of IgG antibody signals in MZ versus DZ twins. Each dot represents a single tested species with the dot-size indicating the IgG signal strength. The line represents the diagonal where X = Y. Density was predictable using the Kernel estimation. Mann–Whitney U tests were used for group comparisons. Error bars in (**a**) represents 95% CI. * indicates statistical significance *p* < 0.05 after adjustment for multiple comparisons. *P. gingivalis* (*i*) refers to strain W381, and (*ii*) refers to strain W83.

Screening for serum IgG antibodies against a panel of oral bacteria revealed signals for all 19 tested species but with varying strengths (Figure 6a and Table S5). The strongest signals were seen for *Aggregatibacter actinomycetemcomitans* and *Haemophilus parainfluenzae* (Figure 6a). Overall the ICC scores were higher for the MZ twins than DZ twins (Figure 6b, *p* = 0.031), with the most marked difference for the two *Porphyromonas gingivalis* and the *Fusobacterium periodonticum* strains (Figure 6b and Table S5).

Heritability was estimated by ACE modeling of the IgG antibody response, and additive genetic variance (A) was statistically significant for 47% (nine of the 19 antibody responses), and among these 67% (six of the nine antibody responses), more than half of the variance could be attributed to A (Figure 6a and Table S5). The additive genetic variance was highest for the IgG response against the two *P gingivalis* strains (0.93 and 0.83, respectively) and the *F. periodonticum* strain (0.76), explaining over 75% of the variance. Component A also explained approximately half of the variance for *Actinomyces odontolyticus, Corynebacterium durum, Corynebacterium matruchotii,* and *Streptococcus oralis* (Figure 6a).

## 4. Discussion

The present study tested for host genetic effects on saliva microbiota taxonomic profiles, associated metabolic functions, and immune responses to oral bacteria in two settings of Swedish twins, i.e., one with young and one with middle-aged twins. Non-shared environmental factors were associated with the presence or absence and abundance of all identified species, while the effects of the host genetic factors were variable at the species level, with strong effects on both the presence and abundance of 27 bacterial species. Among the species affected by additive genetic factors were caries-associated species, such as *Streptococcus mutans*, *Scardovia wiggsiae*, and *Stomatobaculum longum* (a species associated with caries in *S. mutans* infected adolescents [40]). Additive genetic factors were associated with variation in some predicted metabolic pathways in the saliva microbiota, and the pathways under host genetic regulation were enriched for carbohydrate metabolism. Finally, additive genetic influences were most strongly associated with serum antibody response to the periodontitis associated *P. gingivalis*. The study contributed novel insights into the potential genetic driving forces in oral biofilm ecology and host response to oral bacteria, including those that are linked to dental disease, and possibly systemic diseases, in a comparatively large twin study.

In the present study, we aimed to evaluate the impact of host genetic factors on the oral microbiota and the immune response to oral bacteria with the intention to deepen the understanding of the driving forces behind oral biofilm formation and the potential consequences of the oral microbiota. Twin-based studies are an established methodology for heritability research questions that use naturally occurring genetic variations to estimate the relative importance of genetic and environmental factors. For one arm of this study, we chose young twins who were reared together and were still living at home at the time of participation in the study, while for the other arm, we chose twins in later adult life who may have lived apart for many years and were unrelated to the younger twins. The rationale for applying a split-sample design was that for the microbiota characterization, a situation with “native” microbiota was considered desirable and for this, the young twins in the CATSS cohort in the STR offered a group which was likely to have normal saliva flow, healthy mucosal surfaces, and tooth surfaces that were not significantly disrupted by dental restorations, and where saliva had been collected. For the analyses of immune responses, a study group with serum available and with more extended exposure to both caries and periodontal disease conditions were sought. Here the middle-aged twins in the SALT cohort represented an age group where signs of periodontal disease, and hence colonization of periodontal pathogens were likely. Despite this difference in the design and despite using different outcome measures, we observed some similarities in both arms of the study, in particular, that the shared environmental effects were small and that the major differences in concordance were seen between MZ and DZ twins regardless of whether the twins were of the same or opposite sex.

Several studies have examined the gut microbiota in twins and a few targeted the oral microbiota [17,41,42,43]. Several of these studies report environmental factors, such as from diet, drugs, and anthropometric measures, to have major impacts on the shaping of the targeted microbial community [44,45], in keeping with our finding that environmental factors were consistently associated with microbiota taxonomy and function. However, at least for the studies targeting the oral microbiota, detailed comparisons of the genetic influences on the taxonomic profile in general, or of named species are hampered by methodological differences, including differences in targeted 16S rRNA gene regions or even non-DNA-based characterizations, as well as the selection of databases for taxonomic annotation. Where it is possible to make a comparison with other investigations, the results appear broadly consistent with the present study. Especially, three studies that applied 16S rDNA amplicon sequencing of the v3–v4 or the v4 variable regions found more than 50% heritability for several saliva or supragingival tooth biofilm traits [19,20,21].

In line with the gut microbiota [44], we found that twins in the MZ pairs were more similar than twins in the DZ pairs based on qualitative-based (Jaccard) metrics, rather than metrics that were based on quantitative measures (Bray Curtis). This suggests that host genetic factors are more penetrating for community profiles (species detection) than their numbers (temporary abundance). One interpretation may be that quantitative measures are more prone to short-term environmental fluctuations than qualitative measures. In line with this, it has been shown that carriage (detection) of *S. mutans* was stable in the mouth over the years, but the numbers varied significantly over time [46]. Thus, a single measure of species detection may more readily capture phenotypical host traits that affect retention, such as availability of binding receptors or components blocking the bacteria adhesins. Conversely, periodic variations in environmental exposures, such as diet and oral hygiene, may account for variation in the abundance of species at any moment in time and may partially mask genetic effects. Notably, genetic effects may also drive environmental exposure, for example, in people who are genetically predisposed to high intake of sweet foods, and hence have a stable high intake of, e.g., sugar [47,48,49].

The study found that host genetic factors are strongly associated with predicted carbohydrate metabolism in the oral microbiota. This finding is in agreement with a previous study where the heritability of microbial functions proxied by microbial acid formation was estimated at 76% [14]. Here, this is expanded by testing for a wider range of functions and identifying genetic effects on both mono-, di-, and polysaccharide metabolic pathways. The genetic association with carbohydrate metabolism in the oral microbiota likely follows the genetic association of *S. mutans, S. wiggsiae* and other highly acidogenic species but also might reflect genetic effects on a range of more abundant but less acidogenic species. Interestingly, host genetic associations were stronger for several of these species in MZ twins with caries than without. Though not substantiated in this study, this suggests an interaction between the host genetic effect on *S. mutans* and the presence of caries. Possible explanations may include the increased penetrance of genetic effects on *S. mutans* in the presence of environmental features that predispose to caries (gene × environment interaction) or increased penetrance in the presence of genetic risk factors for caries (gene × gene interaction). Notably, heritability for caries is approximately 50% in Swedish twins [7]. However, the findings may also be an artifact of our study design, i.e., if both twins in a pair are at low genetic risk for *S. mutans* colonization, they both end up in the caries-free group, and an effect of genotype is not seen.

Similar to caries, periodontitis is a disease where oral bacteria and host genetic susceptibility both play a pivotal role. Screening of antibody profiles to 19 oral species in serum from adult twins identified a comparably large heritability estimate for the serum levels of antibodies against two strains of *P. gingivalis* and also weaker genetic effects on serum antibodies against *A. actinomycetemcomitans* and *F. alocis*. These species are all associated with periodontal diseases and have been hypothesized to play a role in general inflammatory conditions, possibly by triggering autoimmunity, such as in rheumatic arthritis (RA) [50,51,52,53]. Specifically, *P. gingivalis* has been suggested in RA development by some [53], but not others [54], due to the ability to produce peptidylarginine deiminase leading to protein citrullination in genetically susceptible individuals [55,56,57]. Similarly, the periodontitis-associated *A. actinomycetemcomitans* produces a pore-forming leukotoxin A that triggers the dysregulated activation of citrullinating enzymes in neutrophils and hypercitrullination [58]. The presence of genetic factors which are associated with host immune response to these periodontal pathogens may account for part of the host genetic liability for periodontitis, but also illustrates the way that host genetic variation could act as a confounding feature in studying the relationship between dental and systemic diseases. Study designs which use genetic information to exploit or account for naturally occurring genetic variation may, therefore, be useful in dental epidemiology.

The present study has limitations that need to be acknowledged and considered in the interpretation of the results. In line with all twin based studies, the results do not identify specific genes or genomic regions which regulate the oral microbiota, so they do not provide any information about mechanisms. To address this, genome-wide analysis on oral microbiota taxonomy and function would be needed, similar to those which have been undertaken for the gut microbiota [59]. However, these analyses require very large sample sizes, so they were not considered in the present study. The DNA extraction method did not include additional enzymes to support the disruption of the hard-to-break bacterial cell walls because, at the time of saliva collection, saliva microbiota characterization was not considered. This means that some species may be difficult to detect; however, a validation study comparing taxa profiles and abundances from the method used for the twin samples and the method considered optimal for bacteria DNA release revealed that all species in a known bacteria mixture could be detected by both methods but with systematically lower abundance for some Gram-positive species. The ranking of the relative abundancies was, however, similar to the two methods, as identified by a statistically significant Spearman correlation (mean (range)) rho = 0.66 (0.51 to 0.87). The analysis of taxonomic data was, therefore, restricted to intra-twin comparisons, and we abstained from any analyses beyond that. This may also have affected the power to detect differences in diversity measures based on quantitative measures (Bray Curtis) but not those based on qualitative measures (Jaccard matrix). A further limitation to consider in the data interpretation is the restriction in taxa resolution at the species level using the short v3–v4 16S rDNA sequence for classification. To partly handle this, we used the more stringent 98.5% identity criterium for taxa naming, did not separate species that showed 100% identity by the eHOMING database (i.e., *Streptococcus mitis/Streptococcus oralis/Streptococcus* HMT061), and also reported some of the results at the genus level too.

Finally, a potential cross-reactivity between taxa should be kept in mind when evaluating the checkerboard results. This has been illustrated in a recent publication using the same checkerboard method and set up of bacteria species. That study showed that, among the selected species, the strongest cross-reactivity was among strains of the same species and less between species. Still, it cannot be excluded that shared immune epitopes or non-specific responses could potentially lead to an under- or overestimation of the heritability of the adaptive immune response.

The study also holds some evident strengths, such as its relatively large groups of twins with genetically defined zygosity, and the possibility to evaluate equally sized groups of same and opposite-sex dizygotic twins, the combination of deep Illumina sequencing and taxa classification by a curated database that especially targets oral and upper airway species with taxa resolution to the species level for many species. We believe that the split sample strategy is a strength of the study, as it combined adolescents with normal saliva flow, healthy mucosal surfaces, and mostly non-manipulated tooth surfaces, which may represent a “native” microbiota. In contrast, the older adults are in an age group, which is susceptible to periodontitis and more likely exposed to periodontal pathogens which would be beneficial for antibody screening. In the future, we hope to expand the study of adults by relating the antibody responses to periodontal status and screening a wider panel of bacteria.

Several aspects of the present study are interesting from a clinical perspective. There were strong host genetic effects on several taxa and metabolic functions relevant to caries in the younger age group, and strong host genetic effects on serum antibody levels to known periodontal pathogens in the older age group. In health, the bacterial biofilms are in equilibrium, but the disruption of the balance into a dysbiotic state may lead to permanent loss of tooth tissues (dental caries) or tooth-supporting tissues (periodontal disease). Thus, tooth biofilm architecture is crucial for oral health or disease, but there is also increasing recognition that oral microbiota may be related to systemic diseases [60,61,62]. Understanding the host mechanisms which regulate the oral microbiota composition and function may, therefore, provide novel approaches to treat dental diseases. These mechanisms might involve genetic host regulation of immune response as suggested by the results of the present paper, but also a range of other mechanisms, such as the expression of (glyco) proteins/peptides, which regulate bacteria attachment or metabolism [63] or genetic effects on host behavior such as innate food preferences [15,22,64]. Future studies, including genome-wide host genetic information and oral microbiota data, may help establish these mechanisms.

As anticipated from previous research, the results supported a role of non-shared environmental factors on microbiota composition and function, which is important as lifestyle counseling is a major intervention for dental diseases. While we did not explicitly test for gene-environment interactions in this study, results from sensitivity analysis in caries-concordant groups and results of previous studies suggest that some host genetic factors are more or less important in different situations. Thus, a recent study in a population with high sugar intake, no oral hygiene, rampant caries, and no dental care, all were found to carry *S. mutans* and many also *Streptococcus sobrinus,* whereas in contrasting Swedish adolescents many were *S. mutans*-free and the prevalence of *S. sobrinus* was very low [65]. Therefore, we hypothesize that a genotype that is protective against *S. mutans* in Sweden may not confer the same protection in other settings, and this could be tested through formal interaction analysis. The present results also suggest that genetic factors affect antibody response to a known periodontal pathogen suggested to associate with several general diseased conditions [66], and it may be fruitful to screen a wider panel of bacteria associated with periodontal disease in twins with known periodontal status. This may shed further light on the potential association between periodontitis associated bacteria and general diseases [67].

## 5. Conclusions

The present study supported previous studies in that unshared environmental factors are associated with oral microbiota ecology, but it also identified numerous species where additive genetic influences were strongly associated with being colonized with a bacterial species and the abundance. Species where the detection and abundance were strongly influenced by host genetic factors included caries-associated species, such as *Streptococcus mutans*, *Scardovia wiggsiae* and *Stomatobaculum longum* (a species reported prevalent in caries-affected mutans infected adolescents) [40]. At a microbiota level, there were association between host genetic factors and predicted carbohydrate metabolic pathways. Moreover, the antibody response to the periodontitis and protein citrullinating *P. gingivalis* was associated with additive genetic influences. These findings provide insight into the genetic and molecular basis of oral microbiota development and the stability, and may help understand the consequences of these for oral and general diseases.

## Figures and Tables

**Table 1:** Participant characteristics.

Title	Microbiota Characterization			Antibody Screening		
	MZ	DZ-SS	DZ-OS	MZ	DZ-SS	DZ-OS
Number of twin pairs	71	68	70	70	69	70
Birth year interval	1993–2001	1993–2001	1993–2001	1923–1958	1917–1957	1926–1958
Sampling year interval	2006–2017	2007–2010	2006–2011	2005–2006	2004–2008	2004–2008
Storage year, mean (95% CI)	10.5 (10.2, 10.7)	10.6 (10.4, 10.7)	10.5 (10.3, 10.7)	13.4 (13.3, 13.6)	14.0 (13.8, 14.2)	13.9 (13.7, 14.1)
Sex, % females	49.3	58.8	50.0	65.7	62.3	50.0
Screening age, mean (95% CI)	12.9 (12.5, 13.2)	12.1 (11.7, 12.4)	12.1 (11.7, 12.4)	62.7 (61.4, 64.0)	65.4 (64.0, 66.8)	63.9 (62.5, 65.2)
DMFS, mean (95% CI) ^a^	1.3 (0.6, 2.1)	1.9 (1.2, 2.7)	3.0 (2.2., 3.7)	86.8 (82.4, 91.1)	88.8 (84.5, 93.1)	86.9 (82.6, 91.2)

^a^ DMFS stands for the sum of decayed, missing and filled tooth surfaces. Means are here adjusted for sex and age.

## Supplementary Materials

The following are available online at https://www.mdpi.com/2076-2607/8/8/1126/s1, Table S1: Description of bacteria used in the bacteria mock and checkerboard assay, Table S2: Results from ACE modeling of relative abundances of 267 species, Table S3: Results from ACE modelling of detection of 239 species detected in 5–95 % of the participants, Table S4: Complete list of ICC and ACE values for the 100 top selected functions, Table S5: Screening for serum IgG antibodies against a panel of 19 oral bacteria in 209 adult twin pairs.

## Appendix A

### Estimation of Multiple Testing Burden

Species-level microbial abundance data contain multiple correlated variables organized in a hierarchical cluster pattern [22]. To account for this when adjusting *p*-values for multiple testing, the iPVs package (https://github.com/hughesevoanth/iPVs) was used to estimate the multiple testing burden. First, the species-level standardized abundance dataset was split into two independent datasets each containing one twin in a pair. Next, an iterative hierarchical cluster-cut process was applied to each dataset which a) identifies clusters in the dataset based on a distance matrix and a predefined threshold b) selects a single variable to represent each cluster and then c) iterates until all remaining variables are uncorrelated at the level for the predefined threshold. The number of remaining variables is then taken as an estimate of the multiple testing burden. This method yielded ~180 variables in both the twin 1 and twin 2 datasets with a predefined correlation coefficient threshold of 0.5, so the larger estimate (181, twin 2 dataset) was used for Bonferroni correction.

Estimated microbiota functions and measured plasma antibody responses will also be partially correlated; however, for these traits these is less prior expectation of a hierarchical cluster pattern. For these traits, the estimated number of independent tests was set at the number of principal components, which explained >95% of the variation across all traits, which was 9 for the 100 estimated functions used in ACE modelling and three for the plasma antibody response traits.
